# Common Distribution Patterns of Marsupials Related to Physiographical Diversity in Venezuela

**DOI:** 10.1371/journal.pone.0096714

**Published:** 2014-05-07

**Authors:** Jacint Ventura, Guillem Bagaria, Maria Assumpció Sans-Fuentes, Roger Pérez-Hernández

**Affiliations:** 1 Departament de Biologia Animal, Biologia Vegetal i Ecologia, Facultat de Biociències, Universitat Autònoma de Barcelona, Cerdanyola del Vallès, Spain; 2 CREAF, Cerdanyola del Vallès, Spain; 3 School of Geography and Development, University of Arizona, Tucson, Arizona, United States of America; 4 Departament de Fisiologia Animal, Facultat de Farmàcia, Universitat de Barcelona, Barcelona, Spain; 5 Instituto de Zoología y Ecología Tropical, Facultad de Ciencias, Universidad Central de Venezuela, Caracas, Venezuela; University of Western Ontario, Canada

## Abstract

The aim of this study is to identify significant biotic regions (groups of areas with similar biotas) and biotic elements (groups of taxa with similar distributions) for the marsupial fauna in a part of northern South America using physiographical areas as Operational Geographical Units (OGUs). We considered Venezuela a good model to elucidate this issue because of its high diversity in landscapes and the relatively vast amount of information available on the geographical distribution of marsupial species. Based on the presence-absence of 33 species in 15 physiographical sub-regions (OGUs) we identified Operational Biogeographical Units (OBUs) and chorotypes using a quantitative analysis that tested statistical significance of the resulting groups. Altitudinal and/or climatic trends in the OBUs and chorotypes were studied using a redundancy analysis. The classification method revealed four OBUs. Strong biotic boundaries separated: i) the xerophytic zone of the Continental coast (OBU I); ii) the sub-regions north of the Orinoco River (OBU III and IV); and those south to the river (OBU II). Eleven chorotypes were identified, four of which included a single species with a restricted geographic distribution. As for the other chorotypes, three main common distribution patterns have been inferred: i) species from the Llanos and/or distributed south of the Orinoco River; ii) species exclusively from the Andes; and iii) species that either occur exclusively north of the Orinoco River or that show a wide distribution throughout Venezuela. Mean altitude, evapotranspiration and precipitation of the driest month, and temperature range allowed us to characterize environmentally most of the OBUs and chorotypes obtained.

## Introduction

**Figure 1:**
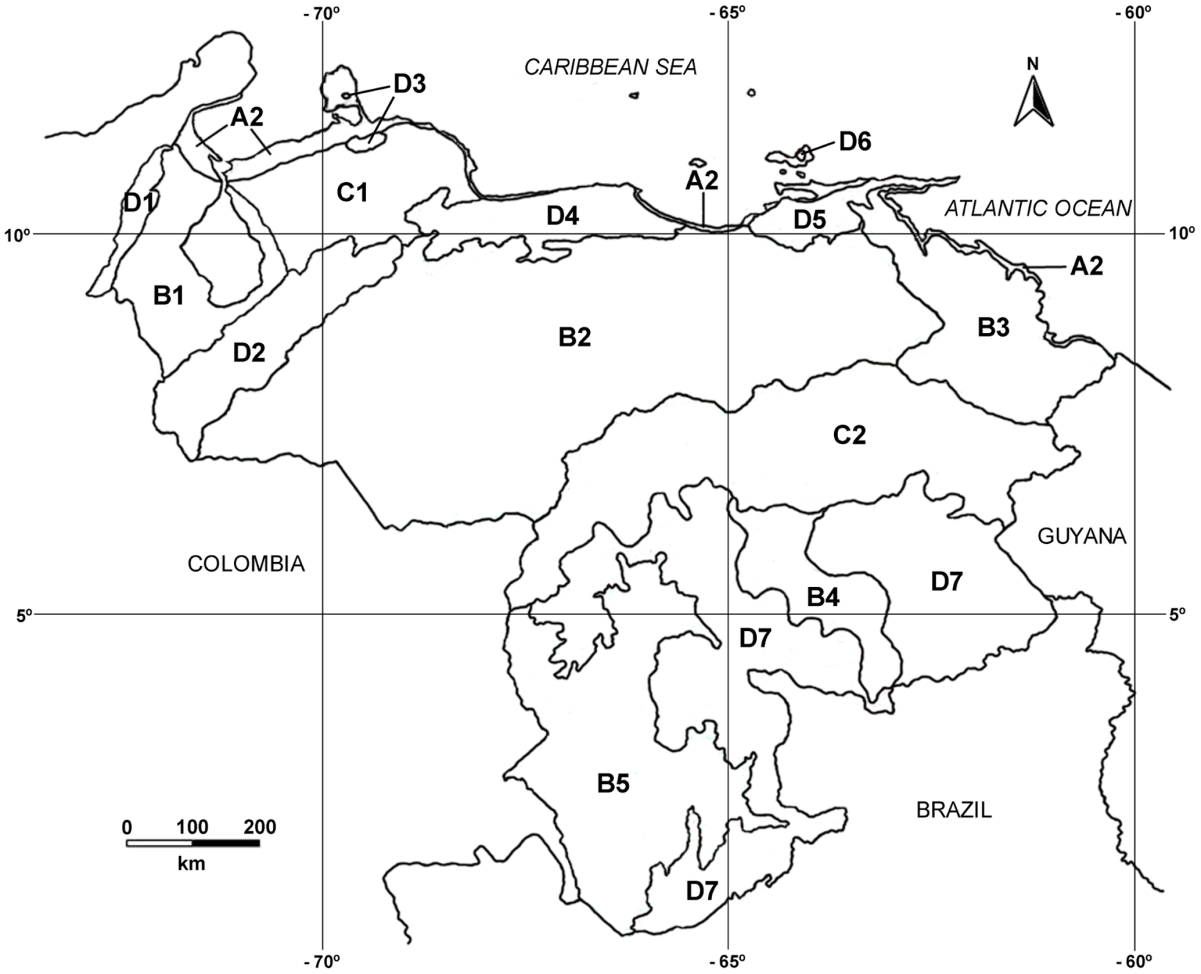
Physiographical sub-regions of Venezuela, according to Huber [5], [6]. A2 Continental coast, B1 Maracaibo depression, B2 The Llanos, B3 Plain of the Orinoco River Delta and the boggy plain of the San Juan River, B4 Peneplain of Caura and Paragua Rivers, B5 Peneplain of the Casiquiare River, Upper Orinoco, C1 Lara-Falcón hill system, C2 Guayana Shield foothill system, D1 Serranía de Perijá, D2 The Andes, D3 Serranía de San Luis and Cerro Santa Ana, D4 Central Coastal Range, D5 Eastern Coastal Range, D6 Cerro Copey, D7 Guayana Massif. Huber's A1 sub-region (Insular coast) was not included in the analyses since there are no marsupials in these extremely dry and small islands.

The current geographic distribution of the fauna in northern South America is strongly linked to dynamic geologic history. In particular, the Andean uplift was a major driver for change in the landscapes and biota in this area and, consequently, was crucial for the evolution of its ecosystems and for the diversification of its species [1], [2]. Within northern South America, Venezuela provides a good example of how this tectonic event, posterior geodynamic processes and variations in the surface geology (e.g., [3], [4] and references therein) have led to a high physiographical diversity. According to Huber [5], [6], the following main landscapes are currently recognized in Venezuela: (A) insular and coastal environments, typical of the Continental coast; (B) lowland plains, found in both the Llanos and the lowland areas along the main Amazonian Rivers; (C) hills and low mountains, which occur in the Lara-Falcón hill system and the Guayana Shield; and (D) high mountains, found either in the ranges or the hills of the Andes (Cordillera de Mérida), Perijá, San Luis, Santa Ana, Central and Eastern Coasts, Copey (in Margarita Island), and Guayana. In turn, these four main landscapes have been divided into different sub-regions (see Fig. 1) identified by geological, climatic and vegetation characteristics [5], [6].

Regarding the mammalian fauna, Venezuela is ranked as the eighth most diverse country in the world [7]. As for marsupials, 34 species of Didelphimorphia and one species of Paucituberculata occur in this zone of the Neotropics [8], representing approximately 30% of the total living species of American opossums. In spite of this rich diversity, information on biogeographical associations of these species in Venezuela is very limited and relates exclusively to observations on the mammal fauna, either in the whole country [9], [10] or in particular geographic systems [4]. In order to detect general patterns of the geographical distribution of mammal species, several approaches have been undertaken using different biogeographical units, such as bioregions [9], regions established on the basis of their “biogeographic and physical-natural” characteristics [10], and zoogeographical units [11], [12]. Nevertheless, in these studies common biogeographical patterns for these species have only been based on comparisons of their distribution areas, without statistical testing. Furthermore, quantitative biogeographical analyses have never been applied to Venezuelan marsupials exclusively.

Methods based on quantitative analyses are the best to establish biogeographical associations, as they provide objectivity and produce consistent and repeatable results. Specifically, this kind of analyses applied to the presence-absence data of several taxa in a number of geographic areas [13] allows for the detection of repeatable biogeographical patterns within the data in the form of biotic elements (groups of taxa with similar distributions) and biotic regions (groups of regions with similar biotas). The quantitative classification method for defining boundaries between ordered locations outlined by McCoy et al. [14] is an extension of the probabilistic similarity techniques [15]. This procedure, complemented by a significance test of the resulting groups (see [16]), constitutes an objective method for measuring the statistical significance of groups obtained by numerical classification (e.g., [17–21].

Venezuela can be considered an excellent model to identify and characterize significant biotic regions and chorotypes regarding its marsupial fauna because of the afore mentioned high physiographical diversity and the availability of a relatively large amount of information on the geographical distribution of the marsupial species. Therefore, the main aim of this study is to determine by quantitative analyses the distribution patterns of the marsupial species in Venezuela, using the physiographical sub-regions defined by Huber [5], [6] as Operational Geographical Units (OGUs; [22]). The specific goals are: (i) to determine significant biotic regions; (ii) to identify associations of species with similar distribution patterns (chorotypes); and (iii) to characterize the biotic regions and the chorotypes using limiting environmental variables for the geographic distribution of these species in Venezuela. To our knowledge, the present study constitutes the first attempt to address these issues for the marsupial fauna of northern South America.

## Materials and Methods

### Study Area and Distributional Data

Venezuela is located in northern South America, between 0°45′ N and 15°40′ N, and between 59°45′ W and 73°25′ W. It covers a total emerged area of 916,445 km^2^. For analytical purposes this study area was divided into 15 OGUs (see Fig. 1) that correspond to the physiographical sub-regions defined by Huber [5], [6].

Information on the distribution of 33 marsupial species in Venezuela (32 belonging to Order Didelphimorphia, Family Didelphidae, and one to Order Paucituberculata, Family Caenolestidae; see Appendix S1 in File S1) was obtained from Pérez-Hernández [23] and Pérez-Hernández et al. [24], and was complemented with data reported in 30 articles published between 1987 and 2013 (see Appendix S2 in File S1). Two species, *Gracilinanus emiliae* and *Gracilinanus agilis*, were excluded from our analyses because distribution information is unreliable. *Gracilinanus emiliae* shows a relatively wide geographic distribution in northern South America (Colombia, Guyana, Surinam, French Guiana and northeastern Brazil), but in Venezuela it has only been found in the Orinoco River Delta, which constitutes a very marginal site within its distribution area [25]. Similarly, *G. agilis* has been reported in two locations that need to be fully corroborated (in forest areas of the Andes and the Central Coastal Range; see [9], [25]–[27]). Additionally, regarding the taxonomic observations by Voss et al. [28], [29], *Marmosops parvidens* has not been included in the analyses, since its presence in the peneplain of the Casiquiare River and Lara-Falcon hill system sub-regions must also be confirmed. Moreover, following Voss et al. [29], we have considered the specimens from the Guayana Massif subregion as *M. pakaraimae*, although initially assigned to *M. parvidens*.

The altitudinal range reported for each species was obtained from the information available in the following collections: Estación Biológica de Rancho Grande (EBRG), Museo de Biología de la Universidad Central de Venezuela (MBUCV), Museo de Historia Natural La Salle (MHNLS) and Colección de Vertebrados de la Universidad de Los Andes (CVULA).

Since several territories in Venezuela remain poorly explored due to their difficult accessibility, the information on the marsupial fauna in some physiographical sub-regions is incomplete. This is the case in certain areas of Serranía de Perijá, piedemonte andino (Andes), Guayana Massif, Orinoco River Delta, and the peneplains of the Caura, Paragua and Casiquiare Rivers. Additionally, it should be noted that our data sources do not allow us to determine the strength of the relationship between the absences of species and the trapping efforts in each sub-region. Nevertheless, despite these drawbacks, it can be assumed that the geographical distribution of the marsupial species in Venezuela is relatively well known (e.g., [8]–[10]). Consequently, the data used in this study are enough to detect reliable general distribution patterns of the marsupial species using physiographical sub-regions as biogeographical units.

### Classification of Physiographical Regions and Species

A presence-absence matrix for the 33 species (Operational Taxonomic Units; OTUs) in the 15 OGUs was constructed (Table 1). The classification of physiographical sub-regions (Q-mode) and species (R-mode) was conducted by using R programming language [30] (see Code S1). Two similarity matrices were obtained (one for each mode) using the Baroni-Urbani and Buser's similarity index (see [31] for details) and implemented in the *simba* package [32]. This pairwise index, when applied to sites, takes into account the number of species unique to each OGU, and both the number of species that co-occur and the number of species that are absent in both OGUs of the same pair. Likewise, when it is applied to species, it takes into account the number of sites unique to each OTU, and both the number of sites where the two OTUs co-occur and the number of sites where the two OTUs are absent. According to Baroni-Urbani and Buser [31], considering common absences is valuable because they highlight differences that are biogeographically informative. Shared absences are, however, multiplied by shared presences in their index to emphasize shared presences and to prevent the possibility that two OGUs would show high similarity because of shared absences alone (see [21] for details).

**Table 1:** Presence (1) or absence (0) of marsupial species in each physiographical sub-region of Venezuela (for abbreviations see Fig. 1).

Species	A2	B1	B2	B3	B4	B5	C1	C2	D1	D2	D3	D4	D5	D6	D7
*Caenolestes fuliginosus*	0	0	0	0	0	0	0	0	0	1	0	0	0	0	0
*Caluromys lanatus*	0	1	0	0	0	1	0	0	1	1	0	0	0	0	0
*Caluromys philander*	0	0	0	0	1	1	0	1	0	0	0	0	0	0	1
*Caluromys trinitatis*	0	0	1	1	0	0	1	0	0	1	0	1	1	1	0
*Chironectes minimus*	0	0	0	1	1	0	0	0	1	1	0	1	1	0	1
*Didelphis imperfect*	0	0	0	0	0	1	0	1	0	0	0	0	0	0	1
*Didelphis marsupialis*	0	1	1	1	1	1	1	1	1	1	1	1	1	0	1
*Didelphis pernigra*	0	0	0	0	0	0	0	0	0	1	0	0	0	0	0
*Gracilinanus dryas*	0	0	0	0	0	0	0	0	0	1	0	0	0	0	0
*Gracilinanus marica*	0	0	0	0	0	0	0	0	1	1	1	1	1	0	0
*Lutreolina crassicaudata*	0	0	1	1	0	0	0	1	0	0	0	0	0	0	1
*Marmosa demerarae*	0	0	0	1	1	1	0	1	0	1	1	1	1	0	1
*Marmosa lepida*	0	0	0	0	1	0	0	0	0	0	0	0	0	0	0
*Marmosa murina*	0	1	0	1	1	1	0	1	1	1	0	1	1	0	1
*Marmosa robinsoni*	0	1	1	1	0	0	1	0	1	1	1	1	1	1	0
*Marmosa tyleriana*	0	0	0	0	0	0	0	0	0	0	0	0	0	0	1
*Marmosa waterhousei*	0	0	0	0	0	0	0	0	0	1	0	0	0	0	0
*Marmosa xerophila*	1	0	0	0	0	0	0	0	0	0	0	0	0	0	0
*Marmosops cracens*	0	0	0	0	0	0	1	0	0	0	0	0	0	0	0
*Marmosops fuscatus*	0	0	0	0	0	0	1	0	1	1	1	1	1	0	0
*Marmosops impavidus*	0	0	0	0	0	0	0	0	0	1	0	0	0	0	0
*Marmosops neblina*	0	0	0	0	0	0	0	0	0	0	0	0	0	0	1
*Marmosops pakaraimae*	0	0	0	0	0	0	0	0	0	0	0	0	0	0	1
*Marmosops pinheiroi*	0	0	0	0	1	0	0	0	0	0	0	0	0	0	1
*Metachirus nudicaudatus*	0	1	1	1	1	1	0	1	1	1	0	0	0	0	1
*Monodelphis adusta*	0	0	0	0	0	0	0	0	0	1	0	0	0	0	0
*Monodelphis brevicaudata*	0	0	0	0	1	1	0	1	0	0	0	0	0	0	1
*Monodelphis palliolata*	0	1	0	1	0	0	1	0	1	1	1	1	1	0	0
*Monodelphis reigi*	0	0	0	0	0	0	0	0	0	0	0	0	0	0	1
*Monodelphis* species A	0	0	1	0	0	0	0	0	0	0	0	0	0	0	0
*Philander andersoni*	0	0	0	0	1	1	0	0	0	0	0	0	0	0	0
*Philander deltae*	0	0	0	1	0	0	0	0	0	0	0	0	0	0	0
*Philander mondolfii*	0	1	1	0	0	0	0	1	1	1	0	0	0	0	1

The relationships between OGUs and OTUs were determined by applying function *hclust* and the unweighted pair-group method using arithmetic averages (UPGMA) from dissimilarity matrices (1-similarity matrices), and assembled into a dendrogram (see [17] for details). We obtained two matrices of significant similarities, one for the physiographical sub-regions and the other for the species, following the table of critical values in Baroni-Urbani and Buser [31]; these matrices reported the similarities that were higher, lower or equal to the similarity value expected at random. The determination of Operational Biogeographical Units (OBUs; see [33]) and chorotypes was accomplished by following the method by McCoy et al. [14] and the approach by Real et al. [16], that consists of testing for the presence of weak or strong significant boundaries between physiographical sub-regions or species. At each node of the corresponding dendrogram, a submatrix that only included the sub-regions or species involved in the node was extracted from the matrix of significant similarities. This submatrix was divided into three zones: A and B, corresponding to the groups separated by the node; and A*B corresponding to the intersection between the former two zones. The number of significant similarities (higher and lower than expected) in each zone was used to compute the parameters DW, DW(A*A), DW(B*B) and DS at each node. DW and DS measure the efficiency of a boundary to separate two groups of physiographical regions or species, whose fauna or geographical distribution, respectively, is similar within but not between each group. DW, which can be separated into DW(A*A) and DW(B*B), measures whether the similarities that are higher than expected tend to be located in zones A and B, but not in A*B. DS measures whether the similarities lower than expected tend to appear in A*B, but not in A or B (see [19] for details). In order to identify significant boundaries, a G-test of independence of the distribution of significant similarities in zones A, B and A*B (after Yates' correction) was conducted for each node of the dendrogram. This test gives the parameter GW for weak segregation and the parameter GS for strong segregation (see [14] for details).

### Relationships between the Biogeographical Units and Chorotypes with Environmental Variables

To detect altitudinal and/or climatic trends in the biotic regions and chorotypes the following variables were considered: mean altitude (MA), mean precipitation of the driest month (MDP), temperature range (TR) and mean evapotranspiration (ME). Both MDP and TR values were obtained from Álvarez Bernal [34], ME values from Legórburu [35] and MA values from Hearn et al. [36]. For each variable, the average in each sub-region was calculated (Table 2). We used only four variables in order to avoid overfitting in the ordination analysis. We selected these variables because we considered them to be limiting for the geographic distribution of the marsupial species in Venezuela.

**Table 2:** Climatic and altitudinal variables for each physiographical sub-region of Venezuela used in the RDA analysis.

Sub-region	MA (m)	MDP (mm)	TR (°C)	ME (mm)
A2	50	8	8	500
B1	61	20	9	1000
B2	150	5	10	1050
B3	51	25	8	1100
B4	202	25	9	1200
B5	642	30	10	1250
C1	406	5	8	750
C2	348	25	9	1100
D1	1561	5	9	1300
D2	1791	25	8	900
D3	529	8	8	800
D4	1066	5	10	900
D5	598	10	8	800
D6	198	5	8	400
D7	855	65	11	1300

MA, mean altitude (in m); MDP, mean precipitation of the driest month (in mm); TR, temperature range, difference between the mean of maximum temperatures of every month and the mean of minimum temperatures of every month (in °C); ME, mean evapotranspiration (in mm).

We conducted an ordination analysis of the species presence-absence table constrained by climatic and altitudinal variables. Since a detrended correspondence analysis (DCA) on the species table resulted in gradient lengths < 2 standard deviation, we used a linear constrained ordination (redundancy analysis, RDA). An ANOVA-like permutation test with 9999 permutations was performed to assess the statistical significance between species composition and environmental variables for each axis. The *vegan* R package [37] was used to perform an RDA analysis and a permutation test.

## Results and Discussion

**Figure 2:**
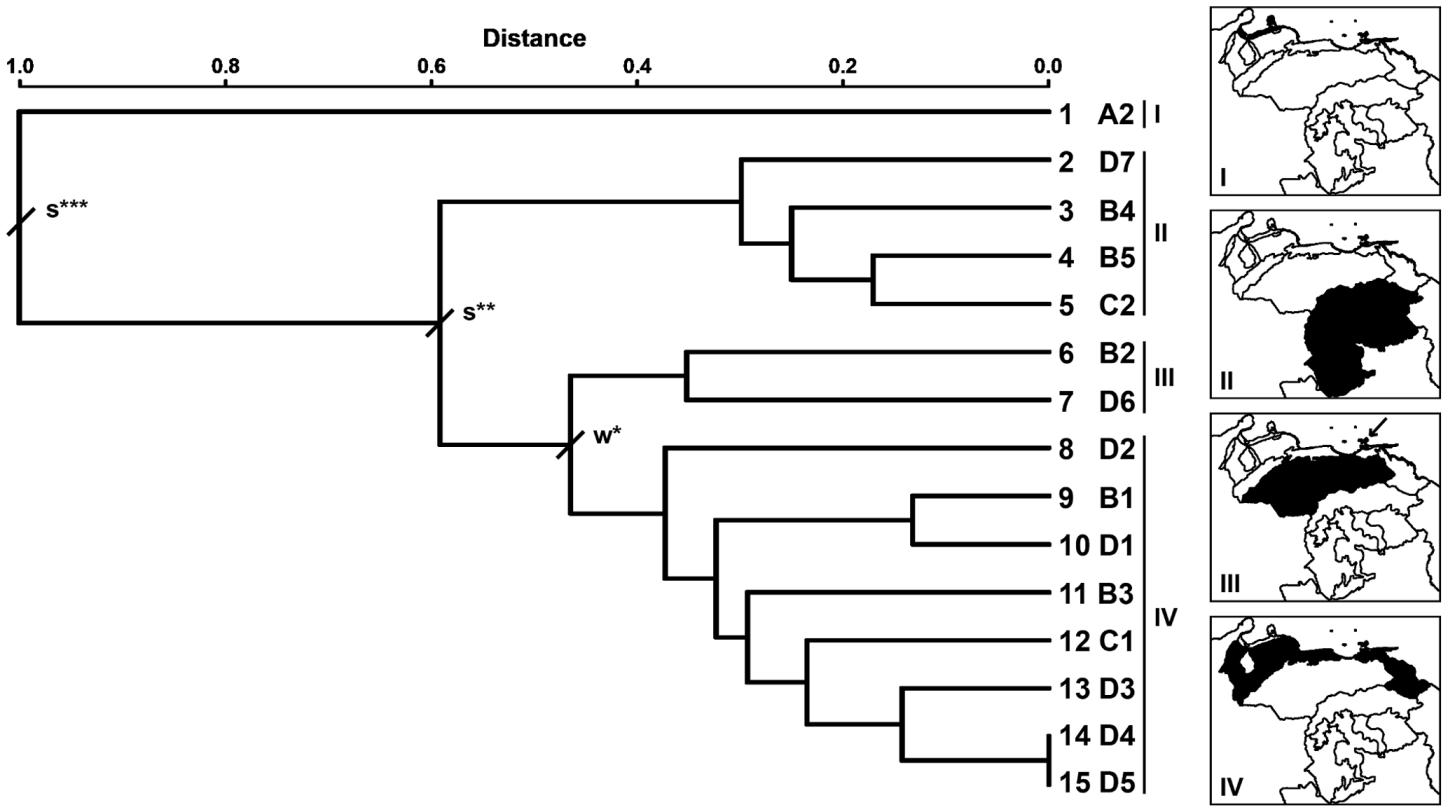
Classification dendrogram of the 15 physiographical sub-regions of Venezuela constructed according to the dissimilarity matrix of marsupial species presence-absence in each sub-region. I–IV, OBUs; w, weak boundary; s, strong boundary; ***P < 0.001; **P < 0.01; *P < 0.05. Maps show the physiographical sub-regions included in each OBU.

**Figure 3:**
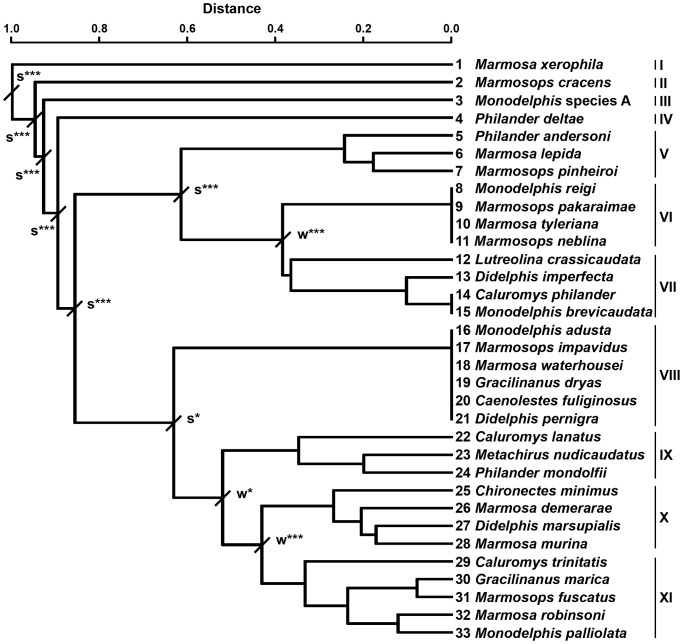
Classification dendrogram of the 33 marsupial species according to their dissimilarity matrix based on presence-absence in each of the 15 physiographical sub-regions of Venezuela. I–XI, chorotypes; w, weak boundary; s, strong boundary; ***P < 0.001; *P < 0.05.

**Figure 4:**
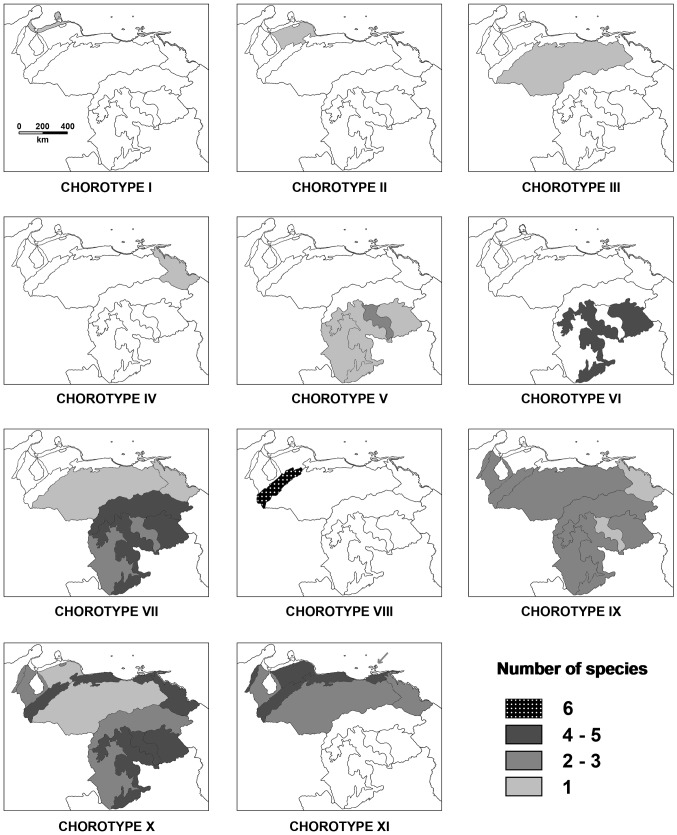
Distribution of the 11 chorotypes of marsupial species in Venezuela. The hatchings indicate the number of species from the chorotype in each sub-region.

The test for the presence of significant boundaries between physiographical sub-regions revealed four OBUs in Venezuela (Table 3). From the species similarity matrix, both the matrix of significant similarities (see Table S1 in File S1) and the corresponding dendrogram were computed (Fig. 2). The test for significant boundaries (Table 3) revealed 11 chorotypes, separated either by weak or strong boundaries (see Table S2 in File S1, and Figs. 3 and 4).

**Table 3:** Significant segregations between the physiographical sub-regions and the species on the dendrogram constructed using UPGMA and on the dissimilarity matrix of marsupial distributions.

	Groups set up by UPGMA			Weak boundary					Strong boundary		
	Group A	Group B	Distance	DW(A*A)	DW(B*B)	DW	GW	P	DS	GS	P
Regions	III	IV	0.464	−0.176	0.343	0.172	5.367	*	−0.116	0.089	n.s.
	II	III–IV	0.592	0.640	0.384	0.512	31.000	***	0.183	8.109	**
	I	II–IV	1.000	0.000	0.157	0.079	4.936	*	0.599	43.482	***
Chorotypes	VI	VII	0.385	0.707	0.416	0.562	16.228	***	0.000	0	n.s.
	X	XI	0.432	0.640	0.515	0.577	19.048	***	0.000	0	n.s.
	IX	X–XI	0.521	0.227	0.221	0.260	4.249	*	−0.071	0	n.s.
	V	VI–VII	0.615	0.252	−0.001	0.277	0.014	n.s.	0.085	17.030	***
	VIII	IX–XI	0.633	0.707	0.207	0.457	37.544	***	0.082	5.681	*
	V–VII	VIII–XI	0.857	0.130	0.147	0.139	64.568	***	0.488	160.889	***
	IV	V–XI	0.896	0.000	−0.220	−0.110	3.997	n.s.	0.178	12.738	***
	III	IV–XI	0.928	0.000	−0.247	−0.123	3.778	n.s.	0.235	21.328	***

GW and GS indicate weak segregation and strong segregation between the groups, respectively. ***P < 0.001; **P < 0.01; *P < 0.05. DW(A*A) and DW(B*B) quantify the internal homogeneity of each group. DW and DS measure the value of each boundary.

**Figure 5:**
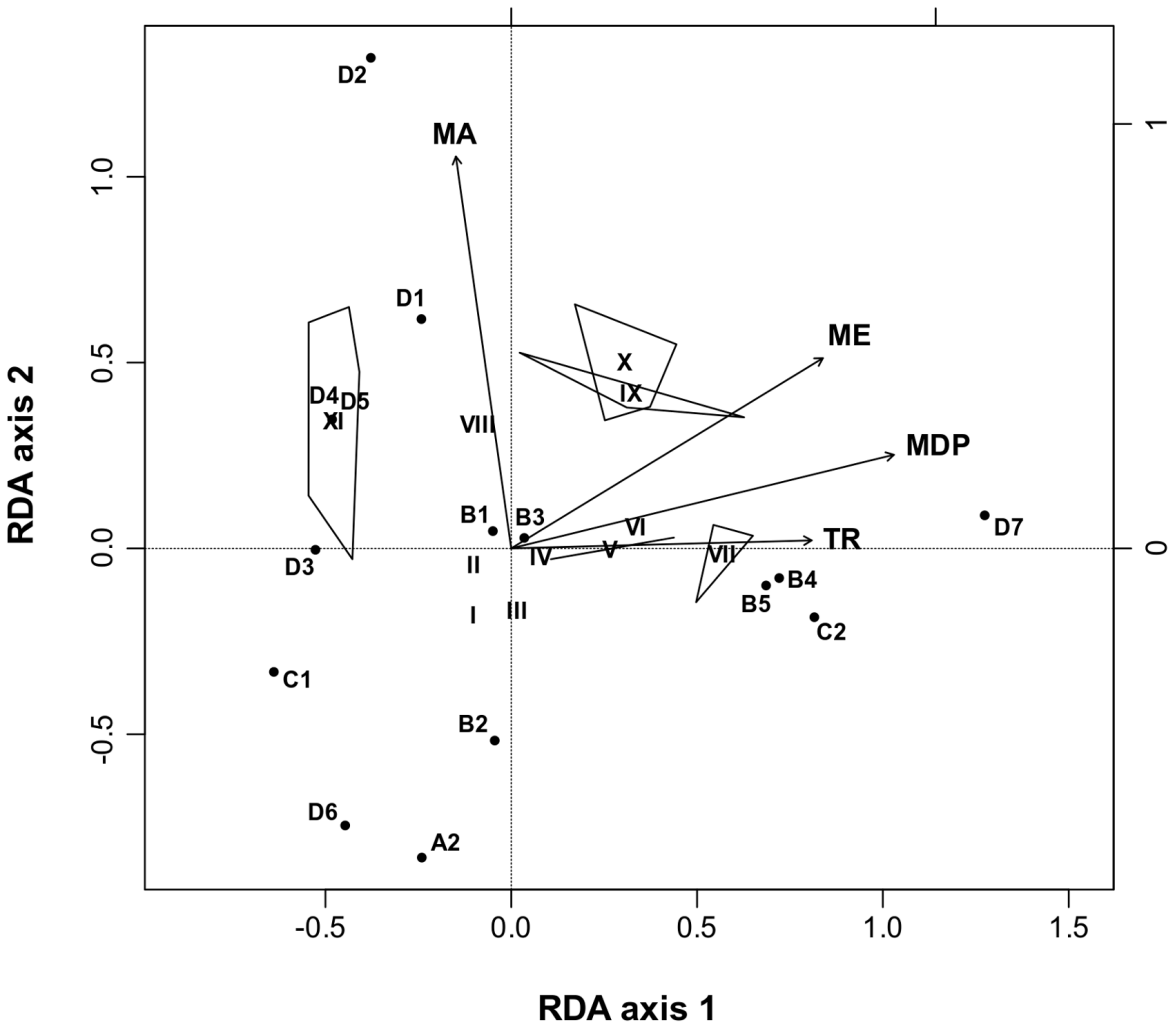
Triplot for the redundancy analysis (RDA) of presence-absence table and environmental variables. MA, mean altitude; MDP, mean precipitation of the driest month; TR, temperature range; ME, mean evapotranspiration. Chorotypes (I–XI) with convex hulls for their species and physiographical sub-regions (OBU I: A2; OBU II: B4, B5, C2, D7; OBU III: B2, B6; OBU IV: B1, B3, C1, D1, D2, D3, D4, D5) are plotted. Only the first and second RDA axes, which explain 25.8% and 16.3% of the total variation, respectively, are shown. Species and regions are scaled symmetrically by the square root of eigenvalues.

In the ordination analysis of the species presence-absence table constrained by environmental variables, the four variables considered jointly explained 49.4% of the total variance (Fig. 5) and the permutation test revealed a significant association only for the first (P < 0.001) and second (P  =  0.002) axes. The first axis explained 25.8% of the variance and had relatively high positive associations with ME, MDP and TR. The second axis explained 16.3% of the variance and was positively associated with MA and, to a lesser extent, with ME.

### Biogeographical Regions

Our Q-mode analysis separated the Continental coast (OBU I) from the other sub-regions by a strong boundary (DS > 0 and GS P < 0.001; Table 3). The Continental coast corresponds entirely to a xerophytic zone, characterized by extremely dry lowlands with high temperatures throughout the year [38]. Redundancy analysis partially corroborated this fact, showing a high association of OBU I with low mean values of altitude and evapotranspiration (Fig. 5). This analysis did not reveal the low MDP as an important constraint, likely because other sub-regions not enclosed in OBU I had similar values for this variable (see Table 2). Only one marsupial species (*Marmosa xerophyla*) occurs in the Continental coast sub-region, because of its dependence on this dry habitat (see below).

A strong boundary (DS > 0 and GS P < 0.01; Table 3) separated OBU II from OBUs III and IV, which in turn were only separated by a very weak boundary (DW > 0 and GW P < 0.05; Table 3). In light of these results and excluding the particular xerophytic zone, Venezuela can be physiographically divided into two main areas that correspond to the territories south (OBU II) and north of the Orinoco River (OBUs III and IV; see Fig. 2). This pattern is concordant with the two main geological regions that are recognized, in general terms, for this country [3]: a southern region that includes Guayana, and a northern region that comprises other mountain ranges, hills and plains.

Specifically, OBU II, which includes the Amazon and Guayana Shield territories (Figure 2), was characterized by a wide TR and relatively high values of both MDP (see also [38]) and ME (Fig. 5). Operational Biogeographical Units III and IV jointly encompassed all the sub-regions located north of the Orinoco River, except A2. In particular, OBU III was composed of the Llanos and Cerro Copey sub-regions, which can be environmentally defined by a low mean altitude and evapotranspiration (Fig. 5). Nevertheless, these sub-regions are physiographically very different. Thus, whereas the Llanos is a vast grassland plain with a bimodal seasonal climate [38], Cerro Copey is a mountain of moderate elevation (960 m.a.s.l.) with a semiarid, or even arid climate, and surrounded by desert plains. In spite of the relatively low altitude of Cerro Copey, in this sub-region there is predominance of green forests and montane grasslands that receive the moisture provided by the trade winds; lower areas are dominated by dry and semideciduous forests. Therefore, the association between the Llanos and Cerro Copey obtained in our Q-mode analysis is not due to a true physiographical relationship, but likely because: i) the only two species found in Cerro Copey (*Caluromys trinitatis* and *Marmosa robinsoni*) also occur in the Llanos; ii) the Llanos shows a relatively low number of species; and iii) the similarity index used in our analysis takes into account shared absences and, although they are multiplied by shared presences, they may influence the association. From the other five species found in the Llanos, one (*Didelphis marsupialis*) has a wide distribution range in Venezuela, three (*Metachirus nudicaudatus*, *Philander mondolfii and Lutreolina crassicaudata*) have a limited and marginal distribution, and one (*Monodelphis* species A) is probably endemic of this sub-region (see below). The pattern obtained for the Llanos concerning the marsupial species composition is concordant with that reported for the non-volant eutherians in Venezuela, i.e. low specific richness, presence of species with a wide distribution and paucity of endemic species (see [10] for details).

Operational Biogeographical Unit IV also showed a very heterogeneous composition. It included the mountain sub-regions of northern Venezuela and two northern flood plains. None of the environmental variables considered was associated with this biogeographical unit. The inclusion in this OBU of such climatically and physiographically diverse sub-regions is likely the result of the wide distribution of many species throughout the north of Venezuela. In fact, most of these zones show a wide range of altitudes, with corresponding variability in temperature and rainfall. In particular, Serranía de Perijá, Andes (Cordillera de Mérida) and the Coastal Ranges show the highest altitudinal range (up to around 5000 m a.s.l.) in Venezuela. In concordance with our results, studies in other mammal groups support the biogeographical affinities between these sub-regions (e.g., [39]–[41]).

The Plain of the Orinoco River Delta, the boggy plain of the San Juan River and the Maracaibo depression are warm and wet areas that show high evapotranspiration mean values. In these sub-regions there are a relatively high number of species that are also present in other physiographical areas of OBU III. In particular, five of these taxa (*Didelphis marsupialis*, *Marmosa murina*, *Marmosa robinsoni*, *Metachirus nudicaudatus* and *Monodelphis palliolata*) occur in both the Maracaibo depression and the Orinoco River Delta in spite of their wide geographic separation. Nevertheless, previous studies revealed that this delta system and the sub-regions south of the Orinoco River show close biogeographical relationships when all mammalian species are considered; in fact, both areas share a relatively high number of species (see [9], [10] for details). Further quantitative analyses using other kinds of OGUs and other environmental variables are needed to elucidate the causes of the particular pattern obtained here for this sub-region.

### Chorotypes

The first four chorotypes (I–IV) comprised only one species and were separated from other groups of species by very strong boundaries (Figs. 3 and 4). None of the variables considered were clearly related these chorotypes (Fig. 5). In particular, chorotype I exclusively included *M. xerophila* and was significantly separated from the other chorotypes due to its very limited geographic distribution and strict habitat requirements (e.g., [42]). This species occurs only in xerophytic zones of the Continental coast sub-region (OBU I) that are characterized by meadows and thickets, and altitudes ranging from 0 to 100 m a.s.l.

Other species with a very restricted distribution is *Marmosops cracens*, the unique species of chorotype II. This taxon is an endemic marsupial of Venezuela that has only been recorded in one locality, in the hills of Falcón State (see [43]).

The single species of chorotype III, *Monodelphis* species A (*sensu* Pine and Handley [44]), occurs exclusively in savanna environments of the Llanos, especially in areas densely covered by grasses at elevations from 20 to 575 m a.s.l. [45]. Interestingly, the taxonomic status of the specimens from these environments attributed to genus *Monodelphis* is controversial (see [44] for details); they have been either assigned to a subspecies of *M. brevicaudata* (*M. brevicaudata orinoci*; e.g., [46]), reported as a full species (*M. orinoci*; e.g., [45], [47]), or considered a taxonomic entity pending specific assignation (*Monodelphis* species A [44]). In this study we followed the latter option because it is based on the most recent and sound revision of this taxonomic problem. If this morphotype is finally recognized as a full species, it will constitute the only known endemic marsupial in the Llanos.

*Philander deltae*, the species of chorotype IV, has been found exclusively in the Orinoco River Delta and the boggy plain of the San Juan River sub-region. In light of the current information, it can be considered the only marsupial species endemic to this zone. The vegetation characteristics of this subregion are peculiar within the Guayana Shield, consisting basically of temporary or permanently flooded forests and palm groves. The forests are generally dense and highly mixed with palms [6]. Among non-volant vertebrates, besides *P. deltae*, only one amphibian (*Hyalinobatrachium mondolfii*; [48], [49]), one reptile (*Anolis deltae*; [50]) and one mammal (*Dasyprocta guamara*; [9], [51]) species have been reported as endemics of this sub-region. As for mammals in particular, the few surveys performed in the Orinoco River Delta determines the relatively poor knowledge of its biodiversity and consequently might be partially related to the scarce number of endemic and also of exclusive species found up to now in this zone (see [8]–[10]).

As for the chorotypes formed by more than one species, a strong boundary separated two main associations of species. In general terms, this boundary segregates the chorotypes formed by species from the Llanos and/or distributed south of the Orinoco River (chorotypes V–VII) from those composed of species that either occur exclusively north of this river or show a wide distribution throughout Venezuela (chorotypes VIII-XI; see Fig. 4).

A strong boundary separated chorotype V from chorotypes VI and VII (Fig. 3). The former comprised three species (*Marmosa lepida*, *Marmosops pinheiroi* and *Philander andersoni*), which occur south of the Guayana Shield foothill system. Although these species jointly cover a wide altitudinal range (*M. lepida*: 63 m a.s.l.; *M. pinheiroi*, 150–1374 m a.s.l.; *P. andersoni*, 0–180 m a.s.l.), the physiographic regions in which they occur are all characterized by a long rainy period throughout the year [38]. In fact, our RDA revealed that the relatively high MDP is associated with chorotype V (Fig. 5). It is worth mentioning that the presence of *M. lepida* in Venezuela is based on a single and relatively recent record [52]; specifically, in the margin of the mouth of the Nichare River (north of the peneplain of the Caura and Paragua Rivers sub-region). In fact, the distribution of *M. lepida* in South America is known from a very low number of records, mainly corresponding to sites below 600 m a.s.l. around the western periphery of the Amazon basin [42].

A weak boundary accounted for the separation between chorotypes VI and VII (Fig. 3). The species included in chorotype VI are found in the Roraima formation, in the Guayana Massif sub-region. Most species of this chorotype occur in premontane and montane habitats in the tepuis (*Marmosops pakaraimae*, 800–1500 m a.s.l., [29]; *Marmosa tyleriana*, 1500–2200 m a.s.l.; *Monodelphis reigi*, 1300–1374 m a.s.l.), the characteristic flat-topped rock formations of this sub-region (Pantepui region [53]); only one species (*Marmosops neblina*) has also been found at lower altitudes in the Roraima formation (140–2000 m a.s.l.). High values of MDP, TR and ME accounted for the distribution of this chorotype (Fig. 5). In fact, the Guayana Massif sub-region is characterized by a very short dry season, a wide temperature range related to the wide altitudinal range of the tepuis and abundant precipitation throughout the year.

The marsupial species richness of the Guayana Massif is clearly higher than that of the other sub-regions of Venezuela, with the exception of the Andes (see Table 1). Three out of the four species of chorotype VI (*M. reigi*, *M. tyleriana* and *M. pakaraimae*) are endemic to this southern zone. This is concordant with results obtained in other vertebrate groups, such as amphibians, reptiles, aves and eutherians, which reveal that the Guayana Massif constitutes an important centre of endemisms [10], [48], [49], [54]–[57]. The fact that the three endemic marsupial species of this physiographical sub-region have been found exclusively at high altitudes in the tepuis seems congruent with the proposal by Señaris and Rojas-Runjaic [49] to recognize the Guayanan highlands as a separate bioregion (“Pantepui” *sensu* these authors) from the other areas of the Guayana Massif. High MDP and ME, and wide TR were related to this chorotype (Fig. 5). In fact, the tepuis region is climatically characterized by a very humid climate and a very short dry season.

Chorotype VII included *Caluromys philander*, *Monodelphis brevicaudata, Didelphis imperfect* and *L. crassicaudata* (Fig. 3). The same environmental variables reported for chorotype VI were also related to the distribution of chorotype VII. The mean altitude did not account for its distribution since their species show a relatively wide altitudinal range although, in general, most of them occur below 1000 m a.s.l. (*C. philander*, 0–600 m a.s.l.; *L. crassicaudata*, 0–940 m a.s.l.; *M. brevicaudata*, 82–1104 m a.s.l.); only *D. imperfecta* markedly surpasses this altitude (0–2550 m a.s.l.). The fact that high values of MDP and ME are associated with the distribution of this chorotype (Fig. 5) evidences the wet characteristics of the physiographical sub-regions where its species occur. *Caluromys philander, D.imperfecta* and *M. brevicaudata* have been found in the physiographical sub-regions south of the Orinoco River. *Lutreolina crassicaudata* has only been reported in four localities, each one belonging to a different physiographical sub-region (Llanos, Plain of the Orinoco River Delta and the boggy plain of the San Juan River, Guayana Shield foothill system, and Guayana Massif). In fact, *L. crassicaudata* is uncommon not only in Venezuela but also in the rest of northern South America, occurring exclusively in grasslands closely associated with aquatic habitats, from flooded areas to small streams [24], [58].

As mentioned above, the other chorotypes included, in general terms, taxa found either exclusively north of the Orinoco River (chorotypes VIII and XI) or widely distributed throughout Venezuela (chorotypes IX and X; Fig. 4). Chorotype VIII was separated by a strong boundary from chorotypes IX–XI and exclusively included Andean species (Fig. 3). Redundancy analysis revealed MA as the only explanatory environmental variable for the distribution of this chorotype (Fig. 5). Specifically, all its species occur between 1000 and 4000 m a.s.l., the latter being the highest altitude reached by a marsupial species (*D. pernigra*) in Venezuela. In this wide range of altitudes, there is high physiographical and climatic heterogeneity, from very warm xerophytic zones (for example, the middle basin of the Chama River [59]) to extremely cold páramo areas at the highest altitudes. This heterogeneity leads, in turn, to diversity in vegetation types (see [60] for details) and to local differences in flora, kinds of soil and hydrology [61].

Regarding the species of chorotype VIII, both *Caenolestes fuliginosus* (2200–2460 m a.s.l.) and *M. impavidus* (2250–2460 m a.s.l.) have been found exclusively in the Páramo Tamá (Estado Táchira), West of the Táchira depression and, therefore, not reaching the Cordillera de Mérida. As for the other species of this chorotype, *Didelphis pernigra* (2150–4000 m a.s.l.), *Gracilinanus dryas* (2210–2410 m a.s.l.) and *Marmosa waterhousei*, which was recently reported in Venezuela, (1000–1200 m a.s.l. [62]), have only been detected in the Cordillera de Mérida, whereas *Monodelphis adusta* (1000–1100 m a.s.l.) occurs in this mountain range and in the west to the Táchira depression. Of all the representatives of this chorotype, only *G. dryas* is endemic to the Venezuelan Andes.

Interestingly, 18 out of the 33 marsupial species studied here were found in the Andes sub-region. When other non-marsupial vertebrate groups are considered, such as amphibians, reptiles [50], [54], [63] or eutherians [10], [40], it is remarkable that this sub-region not only shows a relatively high number of species but also of endemics. The wide altitudinal range and high ecological complexity of the Venezuelan Andes are likely related to this fact [10].

A weak boundary separated chorotypes IX from X and XI (Fig. 3). The former gathered species that globally occur in all physiographical sub-regions, except the Continental coast, Lara-Falcón hill system, Cerro Copey, and the Central and Eastern Coastal Ranges (Fig. 4). High ME and MA environmentally define this group (Fig. 5). The exclusive lack of all species of this chorotype in the xerophytic Continental coast and the surrounding areas of Cerro Copey, two particularly dry sub-regions, can probably explain the implication of ME in the climatic characterization of this chorotype. Species of chorotype IX occur in wide altitudinal ranges (*Caluromys lanatus*: 100–1130 m a.s.l.; *M. nudicaudatus*: 25–2225 m a.s.l.; *P. mondolfii*: 0–1550 m a.s.l.) and are mainly associated with forest environments.

Chorotype X was separated from chorotype XI by a weak boundary (Fig. 3) and its distribution was positively associated with all environmental variables considered (Fig. 5). The joint distribution of the species of chorotype X covers all physiographical sub-regions except the Continental coast and Cerro Copey (Fig. 4). Since a large number of species of this chorotype occur in mountain areas, with high evapotranspiration mean values, this variable shows a high weight in the redundancy analysis (Fig. 5). These species have been found in a wide range of altitudes (*Chironectes minimus*: 0–2100 m a.s.l.; *D. marsupialis*: 0–2550 m a.s.l.; *Marmosa demerarae*: 40–1500 m a.s.l.; *M. murina*: 0–1347 m a.s.l.). Although *D. marsupialis* is a generalist species, this is not the case of the other three taxa. Thus, *M. demerarae* and *M. murina* live in tropical humid forests and are also frequent in second growth and disturbed habitats, and *Chironectes minimus* is a semiaquatic species with a distribution closely tied to tropical forest streams and lakes [64].

Chorotype XI included species with a wide distribution throughout northern and central Venezuela, extending as a whole from the Serranía de Perijá sub-region in the west to the Orinoco River Delta in the east (Fig. 4). Low MDP, TR and ME characterize climatically the distribution of this chorotype (Fig. 5). As shown in the RDA analyses, the variability in mean altitude for this chorotype is very large. Moreover, its species cover a wide altitudinal range, from the lowlands of the Llanos to the high altitudes in the Andes, coast mountains and Serranía de San Luis. Specifically, *M. robinsoni* (0–1160 m a.s.l.) shows a wide distribution north to the Orinoco River, from the Llanos, on the left shore of the Orinoco River, to the northernmost sub-regions of Central and Eastern Coastal Ranges, Cerro Santa Ana, and Cerro Copey. In our dendrogram (Fig. 3), *M. robinsoni* and *C. trinitatis* appear in the same chorotype due to their similar general distribution and, particularly, to their presence on Margarita Island, where they are the only marsupial species reported up to now. *Gracilinanus marica, Marmosops fuscatus* and *M. palliolata* show a very similar distribution in Venezuela. The former is a montane species (0–1750 m a.s.l.) found in the Cordillera de Mérida, Coastal Ranges, Serranía de San Luis and the Llanos of Monagas state. It has been detected in savanna edge habitats and/or in deciduous, humid or cloud forests [65], [66]. The other two species occur in a relatively wide range of altitudes in mountain areas or within their limits (*M. fuscatus*, 0–2232 m a.s.l.; and *M. palliolata*, 0–1500 m a.s.l.).

## Concluding Remarks

This study constitutes the first approach to detect common distribution patterns of marsupials in Venezuela by means of quantitative methods. Specifically, we identified significant biotic regions and chorotypes using physiographical regions as OGUs. In addition, the use of limiting macroclimatic and altitudinal variables allowed us to define OBUs and chorotypes environmentally. It is worth bearing in mind that since the configuration and limits of OBUs and the composition of chorotypes depend on the type of OGUs considered (e.g., [67], [68]), the method applied in the present study constitutes only a specific way to identify biogeographical patterns. Consequently, results have been interpreted exclusively from a physiographical perspective and only very general considerations can be outlined under a historical point of view. Specifically, information on the paleogeographic evolution of the proto-Orinoco and Orinoco rivers from the Paleogene to the present [1], the uplift of the Andes [2], and the recent results based on a time-calibrated molecular phylogeny of the opossums in South America [69] clearly indicate that the biogeographical pattern presented here is posterior to the Late Miocene. Additionally, molecular analyses [69] also suggest that the diversification of opossums in South America (about 17 Ma BP) occurred entirely within moist-forest biomes, with a subsequent invasion of dry-forest areas occurring during or after the Late Miocene (10 Ma, approximately). Undetermined processes during the later Tertiary and Quaternary history of marsupials led to the current distribution of the species of this mammalian group in South America.

In order to understand the current geographic distribution of the marsupial species in this part of South America, additional chorological information and analyses using other OGUs are needed. In particular, future surveys and taxonomic studies should define biotic boundaries, chorotypes and their characterization more accurately. Likewise, further biogeographical analyses based on historical and ecological variables are essential to complement the distribution patterns presented here.

## Supporting Information

Code S1 **R code used to perform the classification of physiographical regions and species from the presence-absence matrix.** (ZIP-archived directory containing an R script calling functions and data).
(ZIP)

File S1 Appendix S1, Nomenclature authorities for all species names mentioned in this paper. Appendix S2, Sources of information on the distribution of the marsupial species in Venezuela. Table S1, Matrix of significant similarities for the physiographical regions in Venezuela. Table S2, Matrix of significant similarities for the marsupial species from Venezuela.
(DOCX)
