# Correction: Common Distribution Patterns of Marsupials Related to Physiographical Diversity in Venezuela

**DOI:** 10.1371/journal.pone.0104466

**Published:** 2014-08-04

**Authors:** The PLOS ONE Staff

The last two lines of Table 3 are missing. Please see the correct Table 3 here.

**Table 3:** Significant segregations between the physiographical sub-regions and the species on the dendrogram constructed using UPGMA and on the dissimilarity matrix of marsupial distributions.

	Groups set up by UPGMA			Weak boundary					Strong boundary		
	Group A	Group B	Distance	DW(A*A)	DW(B*B)	DW	GW	P	DS	GS	P
Regions	III	IV	0.464	-0.176	0.343	0.172	5.367	*	-0.116	0.089	n.s.
	II	III-IV	0.592	0.640	0.384	0.512	31.000	***	0.183	8.109	**
	I	II-IV	1.000	0.000	0.157	0.079	4.936	*	0.599	43.482	***
Chorotypes	VI	VII	0.385	0.707	0.416	0.562	16.228	***	0.000	0	n.s.
	X	XI	0.432	0.640	0.515	0.577	19.048	***	0.000	0	n.s.
	IX	X-XI	0.521	0.227	0.221	0.260	4.249	*	-0.071	0	n.s.
	V	VI-VII	0.615	0.252	-0.001	0.277	0.014	n.s.	0.085	17.030	***
	VIII	IX-XI	0.633	0.707	0.207	0.457	37.544	***	0.082	5.681	*
	V-VII	VIII-XI	0.857	0.130	0.147	0.139	64.568	***	0.488	160.889	***
	IV	V-XI	0.896	0.000	-0.220	-0.110	3.997	n.s.	0.178	12.738	***
	III	IV-XI	0.928	0.000	-0.247	-0.123	3.778	n.s.	0.235	21.328	***
	II	III-XI	0.948	0.000	-0.282	-0.141	3.576	n.s.	0.233	23.658	***
	I	II-XI	1.000	0.000	-0.312	-0.156	3.391	n.s.	0.299	43.706	***

GW and GS indicate weak segregation and strong segregation between the groups, respectively. ***P < 0.001; **P < 0.01; *P < 0.05. DW(A*A) and DW(B*B) quantify the internal homogeneity of each group. DW and DS measure the value of each boundary.
