# Supplementary material for: Common Distribution Patterns of Marsupials Related to Physiographical Diversity in Venezuela
Source: PLoS One. 2014 May 7;9(5):e96714. doi: 10.1371/journal.pone.0096714 (PMC4013023; doi:10.1371/journal.pone.0096714)
Supplement: File S1 — Appendix S1, Nomenclature authorities for all species names mentioned in this paper. Appendix S2, Sources of information on the distribution of the marsupial species in Venezuela. Table S1, Matrix of significant similarities for the physiographical regions in Venezuela. Table S2, Matrix of significant similarities for the marsupial species from Venezuela. (DOCX) [file pone.0096714.s002.docx]

**File S1**

**Appendix S1 Nomenclature authorities for all species names mentioned in this paper.** (*): species used for analyses in this paper.

O. Didelphimorphia

*Caluromys lanatus* (Olfers, 1818) *

*Caluromys philander* (L., 1758) *

*Caluromys trinitatis* (Thomas, 1894) *

*Chironectes minimus* (Zimmermann, 1780) *

*Didelphis imperfecta* Mondolfi and Pérez-Hernández, 1984 *

*Didelphis marsupialis* L., 1758 *

*Didelphis pernigra* J. A. Allen, 1900 *

*Gracilinanus agilis* (Burmeister, 1854)

*Gracilinanus dryas* (Thomas, 1898) *

*Gracilinanus emiliae* (Thomas, 1909)

*Gracilinanus marica* (Thomas, 1898) *

*Lutreolina crassicaudata* (Desmarest, 1804) *

*Marmosa demerarae* (Thomas, 1905) *

*Marmosa lepida* (Thomas, 1888) *

*Marmosa murina* (L., 1758) *

*Marmosa robinsoni* Bangs, 1898 *

*Marmosa tyleriana* Tate, 1931*

*Marmosa waterhousei* (Tomes, 1860) *

*Marmosa xerophila* Handley and Gordon, 1979 *

*Marmosops cracens* (Handley and Gordon, 1979) *

*Marmosops fuscatus* (Thomas, 1896) *

*Marmosops impavidus* (Tschudi, 1845) *

*Marmosops neblina* Gardner, 1990 *

*Marmosops pakaraimae* Voss, Lim, Díaz-Nieto and Jansa (2013)*

*Marmosops pinheiroi* (Pine, 1981) *

*Metachirus nudicaudatus* (É. Geoffroy, 1803) *

*Monodelphis adusta* (Thomas, 1897) *

*Monodelphis brevicaudata* (Erxleben, 1777) *

*Monodelphis palliolata* (Osgood, 1914) *

*Monodelphis reigi* Lew and Pérez-Hernández, 2004 *

*Monodelphis* species A (see Pine and Handley, 2007) *

*Philander andersoni* (Osgood, 1913) *

*Philander deltae* Lew, Pérez-Hernández and Ventura, 2006 *

*Philander mondolfii* Lew, Pérez-Hernández and Ventura, 2006 *

O. Paucituberculata Ameghino, 1894

*Caenolestes fuliginosus* (Tomes, 1863) *

**Appendix S2 Sources of information on the distribution of the marsupial species in Venezuela.**

Anderson RP, Gutiérrez EE, Ochoa J, García FJ, Aguilera M (2012) Faunal nestedness and species-area relationship for small non-volant mammals in “sky island” of northern Venezuela. Stud. Neotrop Fauna Environ 47: 157-170.

Bisbal FJ (1998) Mamíferos de la Península de Paria, estado Sucre, Venezuela y sus relaciones biogeográficas. Interciencia 23: 171-181.

Bisbal FJ, Naveda Rodríguez A (2010) Mamíferos de la cuenca del río Guárico, estados Aragua, Carabobo y Guárico, Venezuela. Mem Fund La Salle Cien Nat 172: 69-89.

Brown BE (2004) Atlas of New World marsupials. Fieldiana Zool 102: 1-308.

Gardner AL (2007) Mammals of South America, Volume 1. The University of Chicago Press, Chicago and London. 669 pp.

Gardner AL, Creighton K (2007) Genus *Marmosops*. In: Gardner AL, editor. Mammals of South America. Chicago and London: The University Chicago Press. pp 61-74.

Guerrero R, Hoogesteijn R, Soriano P (1989) Lista preliminar de los mamíferos del Cerro Marahuaca. T. F. Amazonas, Venezuela. Acta Terramaris 1: 71-77.

Gutiérrez EE, Soriano P, Rossi RV, Murillo JJ, Ochoa J, Aguilera M (2011) Occurrence of *Marmosa waterhousei* in the Venezuelan Andes, with comments on its biogeographic significance. Mammalia 75: 381-386.

Lew D, Pérez-Hernández R (2003) Una nueva especie del género *Monodelphis* (Didelphimorphia:Didelphidae) de la Sierra de Lema, Venezuela. Mem Fund La Salle Cien Nat 159-160: 7-25.

Lew D, Pérez-Hernández R, Ventura J (2006) Two new species of *Philander* (Didelphimorphia, Didelphidae) from northern South America. J Mammal 87: 224-237.

Linares OJ (1997) New locality records of mouse opossums from Venezuela (Marsupialia: Didelphidae). Mammalia 61: 255-259.

Linares OJ (1998) Mamíferos de Venezuela. Caracas: Sociedad Conservacionista Audubon de Venezuela. 691 pp.

Linares OJ, Rivas B (2003) Mamíferos del sistema deltaico (Delta del Orinoco- Golfo de Paria), Venezuela. Mem. Fund. La Salle Cien Nat 159-160: 27-104.

López-Fuster MJ, Pérez-Hernández R, Ventura J, Salazar M (2000) Effect of environment on skull-size variation in *Mamosa robinsoni* in Venezuela. J Mammal 81: 829-837.

López-Fuster MJ, Salazar M, Pérez-Hernández R, Ventura J (2002) Craniometrics of the orange mouse opossum *Marmosa xerophila* (Didelphimorphia: Didelphidae) in Venezuela. Acta theriol 47: 201-209.

Mondolfi E (1997) Lista provisional anotada de los mamíferos de la Cuenca del Río Caura, Venezuela. In: Huber O, Rosales J, editors. Caracas: Ecología de la Cuenca del Río Caura, Venezuela 2. Scientia Guaianae 7. pp. 11-63.

Mustrangi MA, Patton JL (1997) Phylogeography and systematic of the slender mouse opossum *Marmosops* (Marsupialia, Didelphidae). Univ Calif publ zool 130: 1-86.

Ochoa J (2000) Efectos de la extracción de maderas sobre la diversidad de mamíferos pequeños en bosques de tierras bajas de la guayana venezolana. Biotropica 32: 146-164.

Ochoa J, García JF, Caura S, Sánchez J (2009) (“2008”) Mamíferos de la cuenca del río Caura, Venezuela: listado taxonómico y distribución conocida. Mem Fund La Salle Cien Nat 170: 5-80.

Ojasti J, Guerrero R, Hernández O (1992) Mamíferos de la Expedición de Tapirapecó, estado Amazonas, Venezuela. Acta Biol Venez 14: 27-40.

Ramoni-Perazzi P, Bianchi G, Molina M (1994) Hallazgo de la comadreja colicorta *Monodelphis adusta* (Thomas, 1897) en la cuenca del Lago de Maracaibo, Venezuela. Acta Cien Ven 45: 325-326.

Sánchez, J, Lew D (2012) Lista actualizada y comentada de los mamíferos de Venezuela. Mem Fund La Salle Cien Nat 173-174: 173-238.

Soriano PJ (1987) On the presence of the short-tailed oposum *Monodelphis adusta* (Thomas) in Venezuela. Mammalia 51: 321-324.

Ventura J, Lew D, Pérez-Hernández R, López-Fuster MJ (2005) Skull size and shape relationships between Venezuelan *Monodelphis* taxa (Didelphimorphia: Didelphidae), including the recently described species *M. reigi* Lew & Pérez-Hernández 2004. Trop Zool 18: 227-235.

Ventura J, Pérez-Hernández R, López-Fuster MJ (1998) A morphometric assessment of the systematics of *Monodelphis brevicaudata* (Didelphimorphia: Didelphidae) in Venezuela. J Mammal 79: 104-117.

Ventura J, Salazar M, Pérez-Hernández R, López-Fuster MJ (2002) Morphometrics of the genus *Didelphis* (Didelphimorphia: Didelphidae) in Venezuela*.* J Mammal 83: 1087-1096.

Voss RS, Jansa SA (2003) Phylogenetic studies on didelphid marsupials II. Nonmolecular data and new IRBP sequences: Separate and combined analyses of didelphine relationships with denser taxon sampling. Bull Am Mus Nat Hist 276: 1-82.

Voss RS, Jansa SA (2009) Phylogenetic relationships and classification of didelphid marsupials, and extant radiation of New World Metatherian mammals. Bull Am Mus Nat Hist 322: 1-177.

Voss RS, Lim BK, Díaz-Nieto JF, Jansa S (2013) A new species of *Marmosops* (Marsupialia: Didelphidae) from the Pakaraima highlands of Guyana, with remarks on the origin of the endemic Pantepui mammals fauna. Am Mus Novitates 3778: 1-27.

Voss RS, Lunde DP, Simmons NB (2001) Mammals of Paracou, French Guiana: A Neotropical lowland rainforest. Part 2: nonvolant species. Bull Am Mus Nat Hist 263: 1-236.

**Table S1 Matrix of significant similarities for the physiographical regions in Venezuela (for abbreviations see Fig. 1).** +, Values significantly higher than expected at random, P < 0.05; -, values significantly lower than expected at random, P < 0.05; 0, values not different from those expected at random.

|  | D7 | B4 | B5 | C2 | B2 | D6 | D2 | B1 | D1 | B3 | C1 | D3 | D4 | D5 |
| --- | --- | --- | --- | --- | --- | --- | --- | --- | --- | --- | --- | --- | --- | --- |
| A2 | - | - | - | - | - | - | - | - | - | - | - | - | - | - |
| D7 |  | + | 0 | + | 0 | - | 0 | 0 | 0 | 0 | - | - | 0 | 0 |
| B4 |  |  | + | + | 0 | - | 0 | 0 | 0 | 0 | - | 0 | 0 | 0 |
| B5 |  |  |  | + | 0 | - | 0 | 0 | 0 | 0 | - | 0 | 0 | 0 |
| C2 |  |  |  |  | 0 | - | 0 | 0 | 0 | 0 | - | 0 | 0 | 0 |
| B2 |  |  |  |  |  | 0 | 0 | + | 0 | + | 0 | 0 | 0 | 0 |
| D6 |  |  |  |  |  |  | - | 0 | 0 | 0 | + | 0 | 0 | 0 |
| D2 |  |  |  |  |  |  |  | 0 | + | 0 | 0 | 0 | + | + |
| B1 |  |  |  |  |  |  |  |  | + | + | 0 | 0 | 0 | 0 |
| D1 |  |  |  |  |  |  |  |  |  | + | 0 | + | + | + |
| B3 |  |  |  |  |  |  |  |  |  |  | 0 | 0 | + | + |
| C1 |  |  |  |  |  |  |  |  |  |  |  | + | + | + |
| D3 |  |  |  |  |  |  |  |  |  |  |  |  | + | + |
| D4 |  |  |  |  |  |  |  |  |  |  |  |  |  | + |

**Table S2 Matrix of significant similarities for the marsupial species from Venezuela.** +, Values significantly higher than expected at random, P < 0.05; -, values significantly lower than expected at random, P < 0.05; 0, values not different from those expected at random.

|  | *M. cracens* | *M.* species A | *P. deltae* | *P. andersoni* | *M. lepida* | *M. pinheiroi* | *M. reigi* | *M. pakaraimae* | *M. tyleriana* | *M. neblina* | *L. crassicaudata* | *D. imperfecta* | *C. philander* | *M. brevicaudata* | *M. adusta* | *M. impavidus* | *M. waterhousei* | *D. dryyas* | *C. fuliginosus* | *D. pernigra* | *C. lanatus* | *M. nudicaudatus* | *P. mondolfii* | *C. minimus* | *M. demerarae* | *D. marsupialis* | *M. murina* | *C. trinitatis* | *G. marica* | *M. fuscatus* | *M. robinsoni* | *M. palliolata* |
| --- | --- | --- | --- | --- | --- | --- | --- | --- | --- | --- | --- | --- | --- | --- | --- | --- | --- | --- | --- | --- | --- | --- | --- | --- | --- | --- | --- | --- | --- | --- | --- | --- |
| *M. xerophila* | - | - | - | - | - | - | - | - | - | - | - | - | - | - | - | - | - | - | - | - | - | - | - | - | - | - | - | - | - | - | - | - |
| *M. cracens* |  | - | - | - | - | - | - | - | - | - | - | - | - | - | - | - | - | - | - | - | - | - | - | - | - | - | - | 0 | - | 0 | 0 | 0 |
| *M.* species A |  |  | - | - | - | - | - | - | - | - | 0 | - | - | - | - | - | - | - | - | - | - | 0 | 0 | - | - | - | - | 0 | - | - | 0 | - |
| *P. deltae* |  |  |  | - | - | - | - | - | - | - | 0 | - | - | - | - | - | - | - | - | - | - | 0 | - | 0 | 0 | - | 0 | 0 | - | - | 0 | 0 |
| *P. andersoni* |  |  |  |  | + | 0 | - | - | - | - | - | 0 | + | + | - | - | - | - | - | - | 0 | 0 | - | 0 | 0 | 0 | 0 | - | - | - | - | - |
| *M. lepida* |  |  |  |  |  | + | - | - | - | - | - | - | 0 | 0 | - | - | - | - | - | - | - | 0 | - | 0 | 0 | - | 0 | - | - | - | - | - |
| *M. pinheiroi* |  |  |  |  |  |  | + | + | + | + | 0 | 0 | + | + | - | - | - | - | - | - | - | 0 | 0 | 0 | 0 | 0 | 0 | - | - | - | - | - |
| *M. reigi* |  |  |  |  |  |  |  | + | + | + | 0 | 0 | 0 | 0 | - | - | - | - | - | - | - | 0 | 0 | 0 | 0 | - | 0 | - | - | - | - | - |
| *M. pakaraimae* |  |  |  |  |  |  |  |  | + | + | 0 | 0 | 0 | 0 | - | - | - | - | - | - | - | 0 | 0 | 0 | 0 | - | 0 | - | - | - | - | - |
| *M. tyleriana* |  |  |  |  |  |  |  |  |  | + | 0 | 0 | 0 | 0 | - | - | - | - | - | - | - | 0 | 0 | 0 | 0 | - | 0 | - | - | - | - | - |
| *M. neblina* |  |  |  |  |  |  |  |  |  |  | 0 | 0 | 0 | 0 | - | - | - | - | - | - | - | 0 | 0 | 0 | 0 | - | 0 | - | - | - | - | - |
| *L. crassicaudata* |  |  |  |  |  |  |  |  |  |  |  | 0 | 0 | 0 | - | - | - | - | - | - | - | 0 | 0 | 0 | 0 | 0 | 0 | 0 | - | - | 0 | 0 |
| *D. imperfecta* |  |  |  |  |  |  |  |  |  |  |  |  | + | + | - | - | - | - | - | - | 0 | 0 | 0 | 0 | 0 | 0 | 0 | - | - | - | - | - |
| *C. philander* |  |  |  |  |  |  |  |  |  |  |  |  |  | + | - | - | - | - | - | - | 0 | 0 | 0 | 0 | 0 | 0 | 0 | - | - | - | - | - |
| *M. brevicaudata* |  |  |  |  |  |  |  |  |  |  |  |  |  |  | - | - | - | - | - | - | 0 | 0 | 0 | 0 | 0 | 0 | 0 | - | - | - | - | - |
| *M. adusta* |  |  |  |  |  |  |  |  |  |  |  |  |  |  |  | + | + | + | + | + | 0 | 0 | 0 | 0 | 0 | - | 0 | 0 | 0 | 0 | 0 | 0 |
| *M. impavidus* |  |  |  |  |  |  |  |  |  |  |  |  |  |  |  |  | + | + | + | + | 0 | 0 | 0 | 0 | 0 | - | 0 | 0 | 0 | 0 | 0 | 0 |
| *M. waterhousei* |  |  |  |  |  |  |  |  |  |  |  |  |  |  |  |  |  | + | + | + | 0 | 0 | 0 | 0 | 0 | - | 0 | 0 | 0 | 0 | 0 | 0 |
| *G. dryas* |  |  |  |  |  |  |  |  |  |  |  |  |  |  |  |  |  |  | + | + | 0 | 0 | 0 | 0 | 0 | - | 0 | 0 | 0 | 0 | 0 | 0 |
| *C. fuliginosus* |  |  |  |  |  |  |  |  |  |  |  |  |  |  |  |  |  |  |  | + | 0 | 0 | 0 | 0 | 0 | - | 0 | 0 | 0 | 0 | 0 | 0 |
| *D. pernigra* |  |  |  |  |  |  |  |  |  |  |  |  |  |  |  |  |  |  |  |  | 0 | 0 | 0 | 0 | 0 | - | 0 | 0 | 0 | 0 | 0 | 0 |
| *C. lanatus* |  |  |  |  |  |  |  |  |  |  |  |  |  |  |  |  |  |  |  |  |  | 0 | 0 | 0 | 0 | 0 | 0 | 0 | 0 | 0 | 0 | 0 |
| *M. nudicaudatus* |  |  |  |  |  |  |  |  |  |  |  |  |  |  |  |  |  |  |  |  |  |  | + | 0 | 0 | + | + | 0 | 0 | 0 | 0 | 0 |
| *P. mondolfii* |  |  |  |  |  |  |  |  |  |  |  |  |  |  |  |  |  |  |  |  |  |  |  | 0 | 0 | 0 | 0 | 0 | 0 | 0 | 0 | 0 |
| *C. minimus* |  |  |  |  |  |  |  |  |  |  |  |  |  |  |  |  |  |  |  |  |  |  |  |  | + | 0 | + | 0 | 0 | 0 | 0 | 0 |
| *M. demerarae* |  |  |  |  |  |  |  |  |  |  |  |  |  |  |  |  |  |  |  |  |  |  |  |  |  | + | + | 0 | 0 | 0 | 0 | 0 |
| *D. marsupialis* |  |  |  |  |  |  |  |  |  |  |  |  |  |  |  |  |  |  |  |  |  |  |  |  |  |  | + | 0 | 0 | 0 | 0 | 0 |
| *M. murina* |  |  |  |  |  |  |  |  |  |  |  |  |  |  |  |  |  |  |  |  |  |  |  |  |  |  |  | 0 | 0 | 0 | 0 | 0 |
| *C. trinitatis* |  |  |  |  |  |  |  |  |  |  |  |  |  |  |  |  |  |  |  |  |  |  |  |  |  |  |  |  | 0 | 0 | + | 0 |
| *G. marica* |  |  |  |  |  |  |  |  |  |  |  |  |  |  |  |  |  |  |  |  |  |  |  |  |  |  |  |  |  | + | 0 | + |
| *M. fuscatus* |  |  |  |  |  |  |  |  |  |  |  |  |  |  |  |  |  |  |  |  |  |  |  |  |  |  |  |  |  |  | + | + |
| *M. robinsoni* |  |  |  |  |  |  |  |  |  |  |  |  |  |  |  |  |  |  |  |  |  |  |  |  |  |  |  |  |  |  |  | + |
